# Supplementary material for: Causal Relationship between Aging and Anorexia Nervosa: A White-Matter-Microstructure-Mediated Mendelian Randomization Analysis
Source: Biomedicines. 2024 Aug 16;12(8):1874. doi: 10.3390/biomedicines12081874 (PMC11351342; doi:10.3390/biomedicines12081874)
Supplement: Supplementary file 1 [file biomedicines-12-01874-s001.zip › Supplementary Figure/Figure S4/Singe SNP plot of MR analysis for TL to ubm-b-1489.pdf]

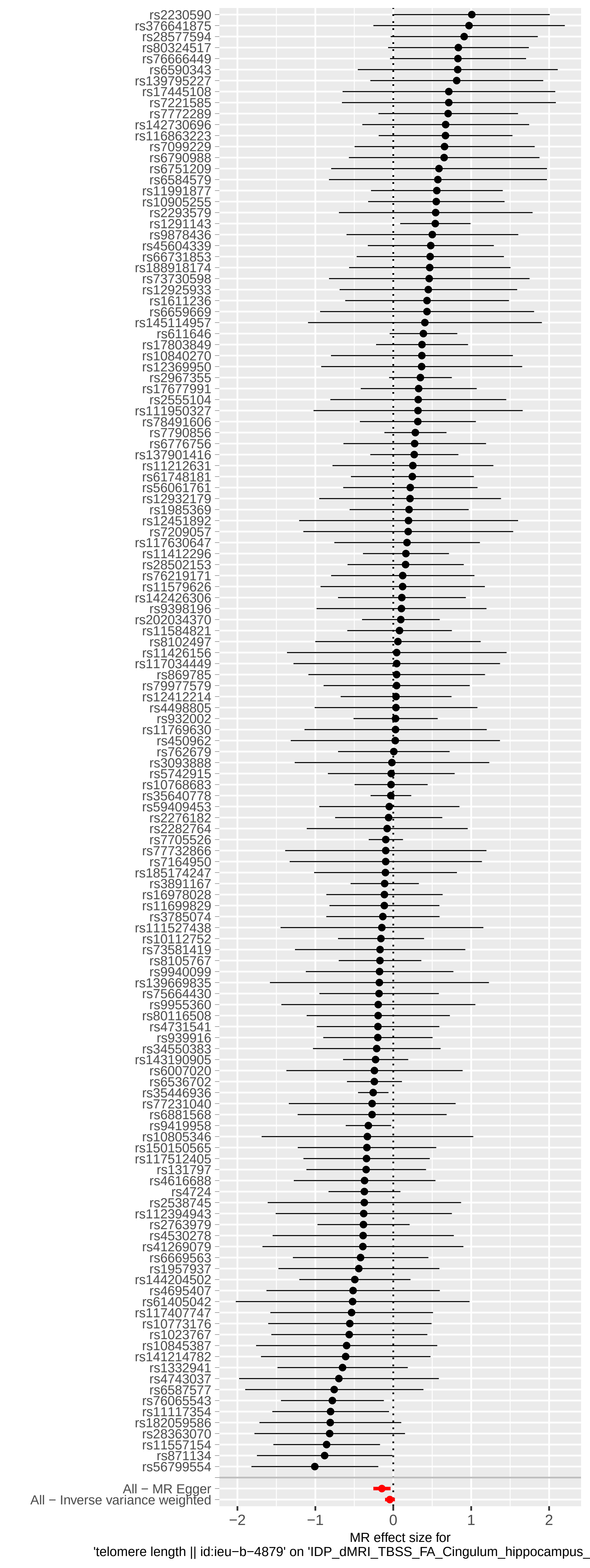
rs2230590
rs376641875
rs28577594
rs80324517
rs76666449
rs6590343
rs139795227
rs17445108
rs7221585
rs7772289
rs142730696
rs116863223
rs7099229
rs6790988
rs6751209
rs6584579
rs11991877
rs10905255
rs2293579
rs1291143
rs9878436
rs45604339
rs66731853
rs188918174
rs73730598
rs12925933
rs1611236
rs6659669
rs145114957
rs611646
rs17803849
rs10840270
rs12369950
rs2967355
rs17677991
rs2555104
rs111950327
rs78491606
rs7790856
rs6776756
rs137901416
rs11212631
rs61748181
rs56061761
rs12932179
rs1985369
rs12451892
rs7209057
rs117630647
rs11412296
rs28502153
rs76219171
rs11579626
rs142426306
rs9398196
rs202034370
rs11584821
rs8102497
rs11426156
rs117034449
rs869785
rs79977579
rs12412214
rs4498805
rs932002
rs11769630
rs450962
rs762679
rs3093888
rs5742915
rs10768683
rs35640778
rs59409453
rs2276182
rs2282764
rs7705526
rs77732866
rs7164950
rs185174247
rs3891167
rs16978028
rs11699829
rs3785074
rs111527438
rs10112752
rs73581419
rs8105767
rs9940099
rs139669835
rs75664430
rs9955360
rs80116508
rs4731541
rs939916
rs34550383
rs143190905
rs6007020
rs6536702
rs35446936
rs77231040
rs6881568
rs9419958
rs10805346
rs150150565
rs117512405
rs131797
rs4616688
rs4724
rs2538745
rs112394943
rs2763979
rs4530278
rs41269079
rs6669563
rs1957937
rs144204502
rs4695407
rs61405042
rs117407747
rs10773176
rs1023767
rs10845387
rs141214782
rs1332941
rs4743037
rs6587577
rs76065543
rs11117354
rs182059586
rs28363070
rs11557154
rs871134
rs56799554
All − MR Egger
All − Inverse variance weighted
−2
−1
0
1
2
MR effect size for
'telomere length || id:ieu−b−4879' on 'IDP_dMRI_TBSS_FA_Cingulum_hippocampus_
